# Frequency of fetal macrosomia and the associated risk factors in pregnancies without gestational diabetes mellitus

**DOI:** 10.11604/pamj.2017.26.62.11440

**Published:** 2017-02-02

**Authors:** Akin Usta, Ceyda Sancakli Usta, Ayla Yildiz, Ruhsen Ozcaglayan, Eylem Sen Dalkiran, Aydin Savkli, Meryem Taskiran

**Affiliations:** 1 Balikesir University School of Medicine, Department of Obstetrics and Gynecology, Turkey; 2 Balikesir Ataturk State Hospital, Clinics of Obstetrics and Gynecology, Turkey; 3 Balikesir University School of Medicine, Department of Internal Medicine, Turkey; 4 Balikesir Ataturk State Hospital, Department of Internal Medicine, Turkey; 5 Balikesir Ataturk State Hospital, Department of Pediatrics, Turkey; 6 Medeniyet University School of Medicine, Department of Obstetrics and Gynecology, Turkey

**Keywords:** Fetal macrosomia, weight gain, gestational diabetes mellitus, body mass index

## Abstract

**Introduction:**

There has been an increased incidence of macrosomic newborns in the world and most of the macrosomic newborns are born from non-GDM pregnant women. The objective of this study was to determine the frequency and the associated risk factors of fetal macrosomia in non-GDM pregnant women.

**Methods:**

A total 4246 consequtive pregnant women who had no GDM was included the study population. Data was collected from hospital database of Balikesir State Hospital between January 2014 and January 2015. Statistical analysis was carried out using the independent samples t-test and chi-squared test. Logistic regression analysis was used to determine the relationships between associated risk factors and the presence of fetal macrosomia. In this analysis, fetal macrosomia was taken as the dependent variable and associated risk factors were taken as independent variables. Results are shown as odds ratios (ORs) (95% CI) in the logistic regression analysis.

**Results:**

366 of the 4246 pregnant women were diagnosed with fetal macrosomia (8.6%). Compared the control women, a statistically significant correlation between fetal macrosomia and pre-pregnancy body mass index (BMI), gestational weight gain (GWG), parity, advanced maternal age, and male fetal sex was found. Maternal BMI, and GWG were the two risk factors most strongly associated with macrosomia.

**Conclusion:**

The prevalance of fetal macrosomia is rising among Turkish women. High pre-pregnancy BMI and GWG represent main modifiable risk factors for macrosomia and need more attention from health care providers.

## Introduction

Fetal macrosomia is defined as a birth weight =4,000 gram or in the 90sup>th percentile for gestational age [1]. Although its prevalence varies among different races and different ethnic groups, it affects approximately 6-10% of all newborns [1, 2]. It is known that fetal macrosomia is associated with a number of maternal and perinatal complications such as infection, postpartum hemorrhage, prolonged labor, high degree perineal tears, cesarean delivery, anesthetic accidents, and thromboembolic events [3]. According the American College of Obstetricians and Gynecology (ACOG) practice bulletin macrosomic fetuses have a greater risk for perinatal asphyxia, meconium aspiration, clavicular fracture, brachial plexus injury, and shoulder dystocia [4]. Furthermore, previous reports have shown that macrosomic infants are at increased risk of developing hypertension, obesity, and type 2 diabetes mellitus in adulthood [5].

Maternal insulin is known to be the primary hormone responsible for intrauterine fetal growth. During pregnancy, irregularity of maternal postprandial blood glucose levels and excessive insuline secretion, especially in the second- and third-trimester can cause fetal macrosomia [6]. The Hyperglycemia and Adverse Pregnancy Outcomes (HAPO) study identified a continuous relationship between maternal glucose and increasing birth weight [7]. A systematic review by Falavigne et al. [8] reported that treatment of gestational diabetes mellitus (GDM) was effective in reducing the rates of macrosomia, preeclampsia, and shoulder dystocia. Therefore, the risk of fetal macrosomia should be considered during prenatal care for pregnant women with pre-gestational diabetes mellitus or GDM. However, approximately 60% of macrosomic fetuses are born to mothers without identifiable risk factors [9]. Unfortunately, ultrasound techniques are not highly reliable in the prediction of macrosomia; probability of correct diagnosis of macrosomia by ultrasound is only 22-37% [10] and macrosomia in non-GDM pregnancies may be overlooked. The aim of prenatal care is prevention and/or accurate identification of fetal macrosomia in order to take precautions against maternal/fetal complications due to macrosomia in pregnant women. The objective of this study was to determine the frequency and the associated risk factors of fetal macrosomia in non-GDM pregnant women.

## Methods

Fetal macrosomia was defined as a birth weight =4,000 grams. Body mass index (BMI) was defined as the mass in kilograms divided by the square of the body height in meters (kg/m^2^) and classified according to the World Health Organization cut-off point[11]: underweight, <18.5 kg/m^2^); normal weight, 18.5-24.9 kg/m^2^); overweight, 25.0-29.9 kg/m^2^); and obese, =30.0 kg/m^2^). GWG was defined as the difference between the measured weight at the last prenatal visit before delivery and the pre-pregnancy weight. Receiver operator characteristic (ROC) curves were formed to detect cut-off values for age, parity and GWG.

GDM was diagnosed with universal screening by a standard two step 50 gram glucose challenge test (GCT). All pregnant women undervent a 50 gram GCT at 24-28 weeks’ gestation. In those with a GCT value of 200 mg/dl or higher GDM was diagnosed. Those with a GCT of =140 - <200 mg/dL underwent a three-hour, 100-gram oral glucose tolerance test (GTT). The diagnosis of GDM was made if two of the four values on the oral glucose tolerance test were abnormal according to the Carpenter and Coustan criteria [12] (0-hours, 95 mg/dl; 1-hours, 180 mg/dl; 2-hours, 155 mg/dl; and 3-hours, 140 mg/dl). Patients who were diagnased with multiple pregnancies, pre-gestational or gestational diabetes mellitus were excluded. In addition, patients who had given bith to a baby before the 20th week of gestational age and those who had given birth to a baby with a birth weight of < 500 grams were excluded from the study. The MedCalc Statistical Software Program version 15.8 (MedCalc, Belgium) was used for statistical analysis. The distribution of all variables in both groups was studied by describing the arithmetic mean ± standard deviation for quantitative data and percentage were used for qualitative data. The independent samples t-test and chi-squared test were carried out for statistical analysis. Logistic regression analysis was used to determine the relationships between associated risk factors and the presence of fetal macrosomia. In this analysis, fetal macrosomia was taken as the dependent variable and associated risk factors were taken as independent variables. Results are shown as odds ratios (ORs) (95% CI) in the logistic regression analysis. A P-value of <0.05 was considered statistically significant for all statistical analyses.

## Results

In this retrospective study, a total of 4246 consecutive non-GDM pregnant women were evaluated between January, 2014 and January, 2015. The characteristics of the mothers with macrosomic and normal newborns are summarized in Table 1. Overall, 366 of all newborns weighed ≥ 4000 grams and the prevalance of macrosomia was 8.6%. Maternal age, parity, pre-pregnancy BMI and gestational weight gain of mothers were significantly higher in macrosomic group than in controls (p=0.0003, p=0.0001, p=0.0001 and p=0.0001, respectively). Gestational age at delivery was similar between the groups (39.1±1.7 and 39.2±1.5 for the macrosomic group and controls, respectively; p=0.2761). Cesarean section and primary cesarean section rates were significantly higher in the macrosomic group than in controls (p=0.0091 and p < 0.0001, respectively). Male newborns made up significantly more of the macrosomic group than the control group (65.6% and 50.2%, respectively; p<0.0001). Weight and height of newborns were significantly higher in the macrosomic group than in controls (3.261±316 and 4.301±241, p<0.0001 and 50.2±1.2, 53.0±1.0, p<0.0001, respectively). Logistic regression analysis of the factors associated with fetal macrosomia rate was carried aut and the factors of age, parity, BMI, GWG and fetal sex were significantly associated with fetal macrosomia rate (data not shown). Thus, all factors were evaluated as independent risk factors into the multivariate models. The ROC analysis showed that fetal macrosomia was significantly higher in women with age ≥ 30, parity ≥ 1 and GWG ≥ 12 in the study population (data not shown). As shown in Table 2, The possibility of having a macrosomic fetus was increased in mothers ≥ 30 years of age (adjusted OR, 1.49; 95% CI: 1.19-1.85), >1 of parity (adjusted OR, 1.76; 95% CI: 1.31-2.35), a pre-pregnancy BMI of ≥ 25 (adjusted OR, 3.35; 95%CI: 2.55-4.40), ≥ 12 of GWG (adjusted OR, 5.45; 95% CI: 3.90-7.61) and male fetal sex (adjusted OR, 1.89; 95% CI: 1.51-2.37).

**Table 1:** Characteristics of mothers with macrosomic and normal newborns

Variables	Control (n=3880)	Macrosomia (n=366)	P- value
**Maternal age**	26.8±5.7	28.0±5.9	0.0003^a^
**Parity.n (%)**			
0	1478 (38.1%)	132 (36.1%)	< 0.0001^b^
1	1999 (51.5%)	172 (47%)	
2	265 (6.8%)	30 (8.2%)	
3	112 (2.9%)	29 (7.9%)	
≥4	26 (0.6%)	3 (0.8%)	
Pre-pregnancy BMI (kg/m^2^)	24.3± 3.5	26.8± 3.6	< 0.0001^a^
GWG (kg)	12.9±5.5	17.1±5.6	< 0.0001^a^
Gestational age at delivery (week)	39.1±1.7	39.2±1.5	0.2761^a^
**Delivery type. n(%)**			
Vaginal delivery	2217(57.1%)	202(55.2%)	0.0091^b^
Cesarean section	1650(42.5%)	162(44.3%)	
Assissted vaginal delivery	13 (0.3%)	2 (0.5%)	
Primary cesarean section. n (%)	440 (11.3%)	87 (23.8%)	< 0.0001^b^
**Newborn sex. n (%)**			
Male	1946 (50.2%)	240 (65.6%)	< 0.0001^b^
Female	1934 (49.8%)	126 (34.4%)	
Newborn weight (kg)	3.261±316	4.301±241	< 0.0001^a^
Newborn Height(cm)	50.2±1.2	53.0±1.0	< 0.0001^a^

Variables mean±SD or n (%)

a independent samples t test.

b chi-squared test

BMI = body mass index; GWG =gestational weight gain

**Table 2:** Risk factors of fetal macrosomia in pregnant women

Variables	n	Macrosomia (%)	aOR (95% CI)	P value
**Maternal age**				
<30	215/2851	7.5%	1.0 (reference)	
≥30	151/1395	10.8%	1.49 (1.19-1.85)	0.0004
**Parity**				
≤1	304/3781	8.0%	1.0 (reference)	
>1	62/465	13.3%	1.76 (1.31-2.35)	0.0001
**Pre-pregnancy BMI (kg/m^2^)**				
Undeweight<18.5	2/153	1.3%	0.29 (0.07-1.23)	0.0938
Normal 18.5-24.9	78/1891	4.1%	1.0 (reference)	
Overweight 25-29.9	185/1539	13.9%	3.17 (2.41-4.17)	<0.0001
Obese ≥30	68/348	19.5%	5.64 (3.98-8.00)	<0.0001
**GWG**				
<12	43/1560	3.0%	1.0 (reference)	
≥12	227/1696	13.8%	5.45(3.90-7.61)	<0.0001
Fetal sex				
Female	126/2060	6.1%	1.0 (reference)	
Male	240/2186	11.0%	1.89(1.51-2.37)	<0.0001

Logistic regression analysis

aOR = adjusted odds ratio; BMI = body mass index; GWG = gestational weight gain

## Discussion

We evaluated fetal macrosomia rate and associated risk factors in non-GDM pregnant women. According to our results, the prevalance of macrosomia was 8.6%, and maternal age, parity, pre-pregnancy BMI, GWG and male fetus rate were significantly higher in macrosomic newborns than in controls. Moreover, we discovered that pre-pergnancy BMI and GWG were the risk factors most strongly associated with fetal macrosomia. The prevalence of fetal macrosomia in pregnant women has been reported as 6%-10% [2] and recent studies have shown increased numbers of fetal macrosomia and infants with birth weight above the 90th percentile for their gestational age in different parts of the world [1]. In some studies performed among Turkish women, macrosomia rates were found to range from 5.2% to 7.6% in the general population [13, 14] and the macrosomia rate was estimated to be 5.9% in non-GDM pregnant women [14]. The prevalence of fetal macrosomia in our study among non-GDM pregnant women living in Aegean territory was 8.6%, which is higher than the prevalences reported in other parts of Turkey. This difference might be associated with different characteristics and scioeconomic status of participants. Previous reports have shown that increased maternal age is associated with fetal macrosomia. A population based study from the United Kingdom reported a 40% increase in the odds of macrosomia in women between 35 and 39 years old in comparison with younger than 35 years old and a 20% increase in risk for women over 40 years old [15]. Recent reports from Turkey have shown that maternal age correlated with neonatal anthropometric measurements and maternal age above 35 years triples the risk of fetal macrosomia [13]. We found that maternal age in the macrosomia group was significantly higher than in controls, and in women older than 30, the risk of fetal macrosomia was 1.5 times higher than in women under 30 years old. According to these the results, maternal age is an important factor in the risk of fetal macrosomia, and our results are consistent with those of previous studies. It is known that metabolic changes occur with increased age, and specific metabolic factor, especially hormonal and endocrine factors, might stimulate higher fetal growth rates among older pregnant women, resulting in higher risk of macrosomic birth [15].

Other factor for the risk of fetal macrosomia is the parity and previous studies have shown that increased parity is associated with higher risk of fetal macrosomia [16, 17]. In a study reported from Sack [17] identified that the frequency of multiparity was higher in mothers with macrosomic newborns than in controls. Dor et al. [16] reported the multiparity rate was approximately 70% in macrosomia group. Similarly the rate of fetal macrosomia in multiparous women has been shown to be 2-3 times higher than that in control group in the majority of studies [18]. In the current study, multiparity rate was approximately 64% among mothers with macrosomic newborns and parity was significantly higher in macrosomia group than in controls. According to results, current report was confirmed with previous reports. Maternal obesity is associated with increased rate of large for gestational age of newborns and fetal macrosomia. The analysis showed that the risk of fetal macrosomia increases with increasing maternal pre-pregnancy BMI. Maternal obesity causes twice the risk of delivery of a macrosomic infants compared to women with normal BMI prior to pregnancy. These results align with other reports that have shown a 1.5-2.3 increase in the adjusted odds of delivering large for gestational age newborns among obese women [2]. We found that pre-pregnancy BMI was significantly higher in macrosomia group than in controls. Moreover, our results identified that, compared the pregnant women had normal BMI, overweight and obese pregnant women were 3.2 and 5.6 increased in the adjusted odds of delivering fetal macrosomia, respectively. According the our results, pregnant women who have = 25 kg/m^2^ for pre-pregnancy BMI tend to have higher risk of fetal macrosomia. Although the optimal GWG is contraversial, previous reports have shown that the rate of GWG is associated with both maternal and fetal health outcomes [19]. Fortner et al. [20] reported that pregnant women gained excessive gestational weight had an almost 2.5 fold risk for hypertensive disorder of pregnancy and a 2.7 fold risk for preeclampsia. The Institute of Medicine (IOM) suggests that pre-pregnancy BMI is a base for determining the optimal GWG range. According to IOM guidelines, before pregnancy underweight women should gain 12.5-18 kg, normal weight women should gain 11.5-16 kg, overweight women should gain 7-11.5 kg, and obese women should gain 5-9.1 kg during the pregnancy [21]. There are no GWG recommendations for pregnant Turkish women. To date, the IOM guidelines are used as the standards of GWG for pregnant Turkish women. However, there are conflicting data related to the IOM guidelines. Wolfe et al. [22] found that pre-pregnancy BMI is not a better predictor of maternal and perinatal morbidity than bodyweight and height alone. Straube et al. [23] found that BMI is not useful as a predictor of weight gain during pregnancy and women with similar pre-pregnancy BMI but different bodyweight and height can differ significantly in GWG during pregnancy. Therefore, the IOM recommendations for GWG based on pre-pregnancy BMI remain controversial. On the other hand, in a recent report by Durie ET al. [24] have shown that 62% of pregnant women have an excessive rate of GWG. A systematic review by Han et al. [25] showed that women with excessive GWG rate (=0.50 kg/week) tend to have macrosomic infants. In our study, we found that GWG was significantly higher in macrosomia group than control and according the our results patients gained = 12 kg during the pregnancy, approximately 5.5 fold increases in the risk of fetal macrosomia. Current study has several limitations. The main limitations were a retrospective nature of the study and relatively small sample of macrosomia group. In addition, the participants were not representative of pregnant women in Turkey, because the participants recruited only from a state hospital in a city in west of our country. Regarding the data collection, accounting for all potential confounding variables is not possible and there was some missing data of pre-pregnancy BMI and GWG even so they were included in the anaysis. We also had no information about maternal and perinatal complication of pregnancies.

## Conclusion

Current report have shown that the prevalance of fetal macrosomia is rising among Turkish women and pre-pergnancy BMI and GWG were preventable risk factors most strongly associated with fetal macrosomia. Pregnant women who have high pre-pregnancy BMI and GWG rate tend to be the risk of fetal macrosomia, particularly for those with ≥ 25 kg/m^2^ of pre-pregnancy BMI and for those with ≥12 kg of GWG. The aim of the prenatal care is prevention and/or accurate identification of fetal macrosomia in order to take precautions against maternal/fetal complications due to macrosomia. Detection of fetal macrosomia before the delivery is one of the important strategies to prevent complications due to macrosomia. However, this strategy does not protect against metabolic risks arising from macrosomia in later life of newborns. Therefore, during the preconception stage provide true information about the preventable risk factors for macrosomia. This is unique strategy for prevention of complications in both mothers and their siblings in all lifetime. Future prospective studies are needed to evaluate the effect of BMI and GWG rate on both pregnancy outcomes among Turkish women.

### What is known about this topic

- There has been an increased incidence of macrosomic newborns in the world;
- Most of the macrosomic newborns are born from non-GDM pregnant women.

### What this study adds

- The prevalance of fetal macrosomia is rising among Turkish women;
- High pre-pregnancy BMI and GWG represent main modifiable risk factors for macrosomia;
- Especially, women with ≥25 kg/m^2^ of pre-pregnancy BMI and for those with ≥12 kg of GWG are under risk for fetal macrosomia.
